# Thiourea-Based H_2_S-Releasing Pramipexole Hybrids as Neuroprotective Agents

**DOI:** 10.3390/antiox15050628

**Published:** 2026-05-15

**Authors:** Angela Corvino, Valentina Citi, Antonia Scognamiglio, Alma Martelli, Vincenzo Calderone, Giulia Neggiani, Carmela Fimognari, Ferdinando Fiorino, Elisa Magli, Rosa Sparaco, Vincenzo Santagada, Giuseppe Caliendo, Beatrice Severino

**Affiliations:** 1 Department of Pharmacy, School of Medicine, University of Naples Federico II, Via D. Montesano, 49, 80131 Naples, Italy; antonia.scognamiglio@unina.it (A.S.); fefiorin@unina.it (F.F.); rosa.sparaco@unina.it (R.S.); santagad@unina.it (V.S.); caliendo@unina.it (G.C.); bseverin@unina.it (B.S.); 2 Department of Pharmacy, University of Pisa, Via Bonanno 6, 56125 Pisa, Italy; valentina.citi@unipi.it (V.C.); alma.martelli@unipi.it (A.M.); vincenzo.calderone@unipi.it (V.C.); 3 Department for Life Quality Studies, Alma Mater Studiorum—Università di Bologna, Corso d’Augusto 237, 47921 Rimini, Italy; giulia.neggiani2@unibo.it (G.N.); carmela.fimognari@unibo.it (C.F.); 4 Department of Public Health, School of Medicine, University of Naples Federico II, Via Panzini, 5, 80131 Naples, Italy; elisa.magli@unina.it

**Keywords:** multitarget agents, pramipexole derivatives, H_2_S-releasing hybrids, thioureas, neuroprotective agents, Parkinson’s diseases (PD), antioxidant compounds, reactive oxygen species (ROS)

## Abstract

Multitarget hybrid molecules are a promising strategy for treating complex neurodegenerative disorders such as Parkinson’s disease (PD), where dopaminergic dysfunction, oxidative stress, neuroinflammation, and cellular senescence coexist and drive disease progression. Here, we developed pramipexole-derived hydrogen sulfide (H_2_S)-releasing hybrids using, for the first time, a thiourea moiety as an H_2_S-donating linker to extend the therapeutic profile of pramipexole beyond dopamine receptor agonism. The hybrids were synthesized and characterized, and their H_2_S-releasing properties were assessed by amperometric and intracellular detection assays. Among the series, compound **2e** (**PRAM-ADA**) showed the most efficient and sustained H_2_S release, indicating a favorable thiol-dependent release profile. **PRAM-ADA** was further evaluated for antioxidant and anti-senescent activities in BV2 microglial cells, as well as for chemical and enzymatic stability under simulated physiological conditions. The hybrid significantly reduced LPS-induced reactive oxygen species accumulation and attenuated oxidative stress–induced cellular senescence, demonstrating a superior cytoprotective profile compared with pramipexole. These findings support the concept that combining dopaminergic activity with controlled H_2_S donation enhances antioxidant and anti-senescent responses, indicating their potential as multitarget agents with neuroprotective properties relevant to neurodegenerative disorders, including PD.

## 1. Introduction

Parkinson’s disease (PD) is a chronic and progressive neurodegenerative disorder characterized by the selective degeneration of dopaminergic neurons in the substantia nigra pars compacta, leading to striatal dopamine depletion and consequent impairment of motor function. Clinically, PD manifests with cardinal motor symptoms, including bradykinesia, resting tremor, rigidity, and postural instability, alongside a wide range of non-motor disturbances such as cognitive decline, depression, sleep disorders, and autonomic dysfunction, all of which significantly impact patients’ quality of life [1,2]. Although current pharmacological treatments, particularly dopamine replacement therapies, effectively alleviate symptoms, they do not halt disease progression and are frequently associated with long-term complications such as motor fluctuations and dyskinesias. This limitation underscores the urgent need for disease-modifying strategies capable of addressing the complex pathophysiology of PD [3].

Accumulating evidence indicates that PD is a multifactorial disorder driven by the interplay of several pathogenic mechanisms, including oxidative stress, mitochondrial dysfunction, neuroinflammation, impaired proteostasis, and glutamate-mediated excitotoxicity, all contributing to progressive neuronal loss [4,5]. The convergence of these processes suggests that targeting a single pathway may be insufficient, thereby supporting the development of multitarget-directed ligands (MTDLs) as a promising therapeutic strategy to simultaneously modulate multiple disease-relevant pathways within a single molecular framework.

In this context, hydrogen sulfide (H_2_S) has emerged as a key endogenous signaling molecule with significant neuroprotective potential. In PD, dysregulation of redox balance, mitochondrial impairment, and chronic neuroinflammation represent central drivers of dopaminergic neurodegeneration, and H_2_S has been shown to modulate all these processes. Specifically, H_2_S contributes to the maintenance of redox homeostasis, supports mitochondrial bioenergetics, regulates protein function through sulfhydration, and suppresses pro-inflammatory signaling cascades [6,7,8]. Consistent with these mechanisms, preclinical studies have demonstrated that H_2_S donors can protect dopaminergic neurons and improve motor outcomes in experimental models of PD [9,10].

Despite these promising findings, the therapeutic translation of H_2_S-based strategies in PD remains limited. Multifunctional compounds capable of combining H_2_S-releasing properties with established pharmacological mechanisms targeting dopaminergic dysfunction and other disease-relevant pathways remain scarce. This gap highlights the need for novel molecular entities that integrate H_2_S donation within a multitarget framework specifically tailored to PD pathology.

Among H_2_S-releasing compounds, isothiocyanates (ITCs) represent an attractive class due to their dual role as bioactive molecules and H_2_S donors. Naturally occurring ITCs, such as sulforaphane, allyl isothiocyanate, benzyl isothiocyanate, and phenethyl isothiocyanate, derived from glucosinolates found in cruciferous vegetables, have demonstrated antioxidant, anti-inflammatory, and neuroprotective effects [11,12,13]. These activities are largely mediated through activation of the Nrf2/ARE pathway, inhibition of NF-κB signaling, and modulation of apoptotic processes, all of which are highly relevant to PD pathogenesis [8,14]. In parallel, synthetic ITCs have been developed to achieve improved control over H_2_S release and pharmacokinetic properties [15,16], and ITC-based derivatives of clinically used drugs, such as memantine and rivastigmine, have shown promising results in neurodegenerative models [17,18], further supporting the applicability of this approach within an MTDL framework.

**Scheme 1:**
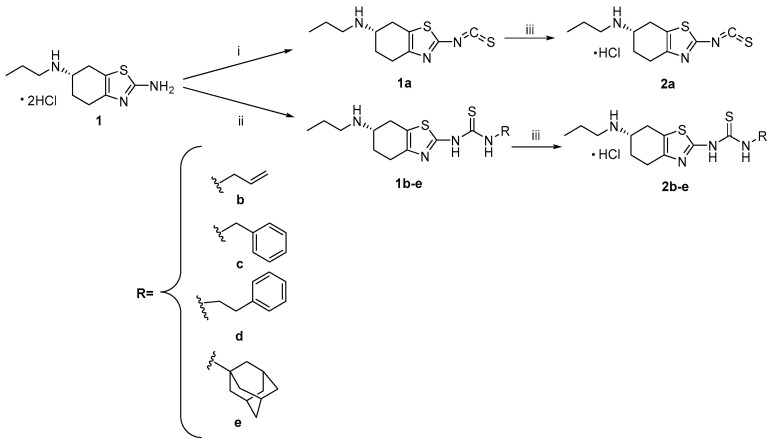
(i) Thiophosgene, Na_2_CO_3_, acetone, 0 °C; (ii) R-NCS, dry acetone, 70 °C, MW; (iii) anhydrous DCM, HCl solution (2.0 M in diethyl ether).

Based on these considerations, we designed and synthesized a novel class of hybrid molecules combining dopaminergic activity with H_2_S-releasing capability. In particular, we selected pramipexole as the core scaffold due to its well-established role as a selective dopamine D_2_/D_3_ receptor agonist used in the treatment of PD [19]. The rationale was to integrate the symptomatic efficacy of dopaminergic stimulation with the disease-modifying potential of H_2_S donation. To this end, a series of pramipexole-based hybrids (compounds **2a**–**e**, Scheme 1) was developed using different design strategies.

In the first approach, the terminal amine of pramipexole was converted into an isothiocyanate group to obtain compound **2a**. In a second-generation design, the pramipexole scaffold was conjugated with natural and synthetic ITCs via a thiourea-based linker (compounds **2b**–**e**). Notably, this linker is known to exhibit intrinsic H_2_S-releasing properties [20,21] and is employed here for the first time as a functional H_2_S-donating element within multitarget hybrid molecules, thereby providing an additional and integrated source of H_2_S release. The natural allyl, benzyl, and phenethyl isothiocyanates were selected on the basis of their established biological activity.

Finally, to further expand the multitarget profile, compound **2e** was designed by incorporating a synthetic ITC derived from amantadine, an NMDA receptor antagonist clinically used in PD for its anti-glutamatergic and anti-dyskinetic effects [22]. This advanced hybrid strategy enables the simultaneous modulation of dopaminergic and glutamatergic neurotransmission alongside H_2_S-mediated cytoprotective mechanisms, thus addressing multiple aspects of PD pathology within a single molecular entity.

## 2. Materials and Methods

### 2.1. Synthesis and Chemical-Physical Characterization of Novel Compounds

Novel multitarget thioureas were prepared starting from commercially available (S)-2-amino-4,5,6,7-tetrahydro-6-(propylamino)benzothiazole dihydrochloride, namely pramipexole (**1**). The synthetic strategy for the preparation of the compounds described here is depicted in Scheme 1.

In particular, the pramipexole isothiocyanate (**1a**) was synthesized in the presence of 3 eq of Na_2_CO_3_ in acetone.

The pramipexole derivatives **1b**–**e** were obtained through the reaction of the primary amine group of pramipexole with specific isothiocyanate reagents. In each case, the two components were directly connected via a thiourea functional group. The reactions were carried out in a microwave oven (DISCOVER 2.0, CEM, Matthews, NC, USA) specifically designed for organic synthesis. Reagents and solvents were placed in a sealed reactor suitable for high-pressure reactions. The synthetic procedure followed a microwave program that included appropriate ramping and holding steps, with the temperature of the stirred reaction mixture monitored using an iWave Sensor (Matthews, NC, USA).

The compounds (**1a**–**e**) were then converted into their corresponding hydrochloride salts (**2a**–**e**), by treatment with diethyl ether saturated with hydrogen chloride gas.

Solutions were concentrated under reduced pressure using a Buchi R-114 rotary evaporator (Buchi Italia S.r.l. Milano, Italy). All reactions were monitored by thin-layer chromatography (TLC) on Merck silica gel 60 F254 (Merk Life Science S.r.l., Milano, Italy) plates, which include a fluorescent indicator. Visualization was achieved under UV light at 254 nm. Preparative chromatographic purifications were performed using silica gel columns (Kieselgel 60, Merk Life Science S.r.l., Milano, Italy). All synthesized compounds were fully characterized by melting point determination, mass spectrometry, and NMR spectroscopy. Melting points were measured with a Buchi Melting Point B-540 apparatus (Buchi Italia S.r.l. Milano, Italy) and obtained from recrystallized or chromatographically purified samples.

Mass spectra of the final products were acquired using an LTQ Orbitrap XL™ Fourier transform mass spectrometer (FTMS) (Thermo Fisher, San Jose, CA, USA), equipped with an ESI ION MAX™M source (Thermo Fisher, San Jose, CA, USA) operating in positive ion mode.

^1^H and ^13^C NMR spectra were recorded at 400 MHz using a Bruker Avance Neo TwoBay spectrometer (Bruker BioSpin Corporation, Billerica, MA, USA), in appropriate deuterated solvents.

### 2.2. Amperometric H_2_S Releasing Properties

The H_2_S-releasing properties of the synthesized pramipexole-based hybrid compounds were assessed using an amperometric method, which enables real-time detection of H_2_S with high sensitivity and selectivity. The experiments were carried out at room temperature using an Apollo-4000 Free Radical Analyzer (WPI, Sarasota, FL, USA) equipped with H_2_S-selective mini-electrodes.

A 10× phosphate-buffered saline (PBS) stock solution was prepared (NaH_2_PO_4_·H_2_O 1.28 g, Na_2_HPO_4_·12H_2_O 5.97 g, NaCl 43.88 g in 500 mL of distilled water) and stored at 4 °C. Before each experiment, the buffer was diluted 1:10 with distilled water to obtain the assay buffer (AB), and the pH was adjusted to 7.4.

The mini electrode was first equilibrated in 2 mL of AB until a stable baseline was achieved. Each test compound was dissolved in DMSO and added directly to the AB to reach a final concentration of 1 mM (DMSO 1%). The tested compounds included **1**, **2a**, **2b**, **2c**, **2d**, and **2e**.

The assay was performed both in the absence and in the presence of L-cysteine (4 mM), whose thiol group mimics intracellular free thiols. In the latter condition, L-cysteine was added to the AB immediately before compound addition to better reflect physiologically relevant environments.

H_2_S release was continuously monitored for 30 min, and the corresponding amperometric signals (pA) were recorded. Calibration curves were generated using known concentrations of NaHS (1, 3, 5, and 10 μM) in acid buffer (pH 4.0) to convert current readings into H_2_S concentrations (μM). The resulting H_2_S concentrations were plotted over time to evaluate and compare the H_2_S-releasing behavior of each compound.

### 2.3. In Vitro Evaluation

#### 2.3.1. Cell Culture

BV-2 murine microglial immortalized cells (Cytion, Heidelberg, Germany) were cultured in RPMI 1640 medium (R0883, Sigma-Aldrich^®^, now Merck KGaA, Darmstadt, Germany) supplemented with 10% fetal bovine serum (FBS), 1% L-glutamine, and 1% penicillin–streptomycin solution (100 U/mL and 100 µg/mL, respectively; all from Sigma-Aldrich^®^). Cells were grown in T-75 flasks at 37 °C in a humidified 5% CO_2_ atmosphere until they reached approximately 90–95% confluence before starting the experiments and were used up to passage 20.

#### 2.3.2. Measurement of Intracellular H_2_S Release in Murine Microglial Cells

BV-2 cells were seeded at a density of 72,000 cells per well in a black 96-well plate. After 24 h, the culture medium was replaced with 180 µL per well of a freshly prepared WSP-1 working solution (Washington State Probe-1, 3-methoxy-3-oxo-3H-spiro[isobenzofuran-1,9-xanthen]-6-yl-2(pyridin-2-yldisulfanyl)benzoate; Cayman Chemical, Ann Arbor, MI, USA) at a final concentration of 100 µM. WSP-1 is a highly specific fluorescent probe for the detection of H_2_S, which undergoes cyclization upon reaction with intracellular H_2_S, leading to the release of a fluorophore (benzodithiolone) with λ ex/em = 476/516 nm.

Cells were incubated with the WSP-1 working solution for 30 min at 37 °C and 5% CO_2_, protected from light, to allow intracellular uptake of the dye. In parallel, wells without cells (“blank” wells) were filled with the same WSP-1 solution and treated with identical conditions to evaluate potential background fluorescence.

After incubation, the WSP-1 solution was removed and replaced with 180 µL of standard assay buffer (20 mM HEPES, 2 mM KCl, 120 mM NaCl, 1 mM MgCl_2_·6H_2_O, 5 mM glucose, 2 mM CaCl_2_·2H_2_O, pH 7.4).

A basal fluorescence reading was then recorded using a multimode plate reader (EnSpire^®^, PerkinElmer, Waltham, MA, USA), reflecting endogenous intracellular H_2_S levels.

Then, BV-2 cells were treated in triplicate with 20 µL of H_2_S donor compounds at final concentrations of 300 µM and 100 µM. Cells were also exposed to the vehicle (DMSO 1%), and to diallyl disulfide (DADS; Merck KGaA, Darmstadt, Germany) 100 µM, used as a positive control due to its known properties as a slow-releasing H_2_S donor [23].

Fluorescence increase (Fluorescence Index, FI), resulting from the reaction between WSP-1 and H_2_S released by the donor compounds, was monitored for 60 min by acquiring fluorescence measurements every 5 min.

Furthermore, the safety profile of the compounds was evaluated as reported in Supporting Information (Figure S1).

For data analysis, background fluorescence from blank wells was subtracted from the corresponding values in treated wells. The resulting time-course fluorescence data were then used to calculate the area under the curve (AUC).

#### 2.3.3. Antioxidant Effect in an LPS-Induced BV-2 Model

The antioxidant effect of the pramipexole hydrochloride derivative **2e** was evaluated in comparison with pramipexole hydrochloride (**1**) by assessing intracellular production of reactive oxygen species (ROS). ROS levels were quantified using the Muse^®^ Oxidative Stress Kit (Luminex Corporation, Austin, TX, USA), following the manufacturer’s instructions. This assay enables the identification and quantification of ROS (+) cells by detecting superoxide anions via a fluorescence-based method, using the minicytofluorometer Muse^®^ Cell Analyzer (Luminex Corporation).

BV-2 murine microglial cells were seeded in 6-well plates at a density of 5 × 10^5^ cells/well and incubated for 24 h at 37 °C in a humidified 5% CO_2_ atmosphere. After incubation, the medium was replaced with 980 µL of fresh RPMI and cells were then pre-treated with **2e** (0.3 and 1 µM) or **1** (0.3 and 1 µM). All compounds were solubilized in DMSO and diluted in culture medium to obtain a final DMSO concentration of 0.1%. After 1 h of incubation, LPS 10 µg/mL was added for 24 h to induce oxidative stress.

Then, cells were detached using a scraper, centrifuged at 1200 rpm for 5 min, and the pellets were resuspended in 500 µL of 1× Assay Buffer (Muse^®^ Oxidative Stress Kit), adjusting the final cell density to 1 × 10^6^–1 × 10^7^ cells/mL as recommended by the manufacturer.

The Muse^®^ Oxidative Stress Reagent was diluted 1:100 in 1× Assay Buffer to obtain the intermediate solution, which was further diluted 1:80 to prepare the working solution. A 10 µL aliquot of the cell suspension was mixed with 190 µL of working solution, gently vortexed for 3–5 s, and incubated at 37 °C in the dark for 30 min. Samples were then briefly mixed again and analyzed using the Muse^®^ Cell Analyzer (Luminex).

The assay distinguishes ROS-negative [ROS (−)] cells from ROS-positive [ROS (+)] cells (oxidatively stressed cells). Results were expressed as the percentage of ROS (+) and ROS (−) cells within the total population. At the end of the experimental procedure, cell viability was also assessed (Supporting Information, Figure S2).

#### 2.3.4. Evaluation of Anti-Senescence Effect in BV-2 Cells

To establish an in vitro model of microglial senescence, BV-2 murine microglial cells were exposed to oxidative stress induced by H_2_O_2_. Senescence was assessed by detecting senescence-associated β-galactosidase (SA-β-gal) activity, a lysosomal enzyme widely used as a biomarker of cellular senescence due to its elevated expression in senescent cells.

BV-2 cells were seeded in 6-well plates (75,000 cells/well) pre-coated with 1% gelatin and incubated at 37 °C in a humidified 5% CO_2_ atmosphere. Gelatin coating was applied to improve BV2 cell adherence, in order to prevent cell detachment and ensure maintenance of a stable monolayer throughout the procedures. After 24 h, the medium was replaced with 900 µL of fresh medium. Oxidative stress was induced by adding 100 µL of H_2_O_2_ to reach final concentrations of 50, 100, or 200 µM. Control wells received 100 µL of medium without H_2_O_2_. After 3 h, the medium was replaced with 2 mL of complete medium, and cells were incubated for 72 h.

Before staining, the citrate/phosphate buffer was prepared (Na_2_HPO_4_ 756 mg, C_6_H_8_O_7_ 1720 mg in 200 mL of bidistilled water) and stored at 4 °C. On the day of the experiment, the pH of the citrate/phosphate buffer was adjusted to 5.6, and the staining solution was freshly prepared using this buffer (K_3_[Fe(CN)_6_] 5 mM, K_4_[Fe(CN)_6_]·3H_2_O 5 mM, MgCl_2_ 2 mM, NaCl 150 mM in 10 mL of citrate/phosphate buffer). The G/F fixative (1.85% formaldehyde and 0.2% glutaraldehyde in 1× PBS) was also freshly prepared on the same day.

Following 72 h of incubation, cells were fixed with 500 µL of freshly prepared G/F fixative for 5 min at room temperature. During fixation, the X-gal stock solution (50 mg/mL in DMSO) was added to the staining solution to reach a final concentration of 1 mg/mL. After fixation, cells were washed once with 1× PBS and incubated with 500 µL of the freshly prepared staining solution for 3 h at 37 °C on a temperature-controlled plate shaker in the dark. X-gal (5-bromo-4-chloro-3-indolyl-β-D-galactopyranoside) is a sensitive chromogenic substrate for β-galactosidase; upon hydrolysis by SA-β-gal, it produces an insoluble blue compound (5,5′-dibromo-4,4′-dichloro-indigo) that accumulates in senescent cells, enabling their visual identification [24].

After staining, the solution was removed, cells were washed with PBS, and 500 µL of isopropanol was added for 15 min to preserve staining. Bright-field images were acquired using a light microscope, capturing at least three representative fields per well. Quantification was performed in blinded analysis using ImageJ software (version 1.54, NIH, Bethesda, MD, USA). For each microscopic field, the percentage of blue-stained area (SA-β-gal positive) was determined in relation to the total area observed. At the end of the experimental procedure, cell viability was also assessed (Supporting Information, Figure S3).

The potential anti-senescent effect of the H_2_S-releasing pramipexole derivative **2e** was evaluated in comparison with pramipexole hydrochloride (**1**). BV-2 cells were pre-treated with the compounds prior to oxidative stress induction by H_2_O_2_, following the senescence protocol described previously.

Cells were treated with **1** or **2e** at final concentrations of 0.3 µM and 1 µM for 1 h. After incubation, oxidative stress was induced by adding 100 µL of H_2_O_2_ to reach a final concentration of 50 µM. Untreated cells served as negative controls, while cells treated with H_2_O_2_ alone were used as positive controls for senescence induction.

After 3 h of H_2_O_2_ exposure, the medium was replaced with fresh complete medium, and cells were incubated for 72 h. The subsequent steps, including fixation, SA-β-gal staining, and image acquisition, were carried out.

#### 2.3.5. Data Analysis

One-way ANOVA followed by Bonferroni’s post hoc test was used to compare the means of three or more treatment conditions, using GraphPad Prism 8 (GraphPad Software). The results are reported as the mean ± SEM. *p*-value was considered statistically significant at *p* < 0.05 (*), *p* < 0.01 (**), *p* < 0.001 (***), or *p* < 0.0001 (****). Each experiment was performed in triplicate and repeated independently at least twice (n ≥ 6).

### 2.4. Chemical and Enzymatic Stability

The chemical and enzymatic stability of the tested compound was evaluated under different simulated physiological conditions. All experiments were performed at 37 ± 0.5 °C, and analyses were conducted in triplicate.

#### 2.4.1. Stability in PBS and SGF

For chemical stability studies, a stock solution of tested compound (10 mM) was prepared in DMSO and diluted with the appropriate preheated buffer to obtain a final concentration of 100 µM, maintaining a final DMSO content below 1% (*v*/*v*). Stability at physiological pH was assessed in phosphate-buffered saline (PBS, pH 7.4), while acidic stability was evaluated in simulated gastric fluid (SGF without pepsin, 2.0 g/L sodium chloride and 2.917 g/L HCl, pH 1.2). PBS buffer was prepared by dissolving one tablet of Phosphate Buffer Saline (Invitrogen, Waltham, MA, USA) in 100 mL of distilled water and adjusting pH if necessary. The resulting buffer contains 10 mM phosphate and 150 mM sodium chloride.

Samples were incubated at 37 °C for 24 h, and at predetermined time points, aliquots were withdrawn and analyzed with RP-HPLC, using a validated analytical method. The percentage of intact compound was plotted as a function of time, and the half-life (t_1_/_2_) was calculated assuming first-order degradation kinetics.

For the calibration curves, standard solutions (r2 > 0.99) in PBS or SGF, over the concentration range of 0.2–250 μM, were used. HPLC analyses were performed as described below.

#### 2.4.2. Stability in DMEM

Enzymatic stability was assessed by incubating the tested compound in Dulbecco’s Modified Eagle Medium (DMEM) supplemented with 1% L-glutamine, 1% Pen-Strep, 10% Fetal Bovine Serum (FBS). A solution of compound **2e** (10 mM) in DMSO was added to the medium to a final concentration of 200 µM, and the mixture was incubated at 37 °C. At selected time points, 200 μL of each aliquot was collected and added to 200 μL of CH_3_CN containing 0.1% of TFA to deproteinize the medium. The samples were vortexed using a Velp ZX3 Vortex mixer (VELP, Usmate Velate, Italy) and then centrifuged for 10 min at 15,000 rpm using Eppendorf Centrifuge 5425 (Eppendorf, Hamburg, Germany). Each clear supernatant was collected and filtered by 0.45 μm PTFE filters (Macherey-Nagel, Düren, Germany). 20 μL aliquots were withdrawn and analyzed by RP-HPLC, as reported below. The experiment was performed in triplicate, and the percentage of intact compound was plotted as a function of time, and the half-life (t_1_/_2_) was calculated assuming first-order degradation kinetics.

For the calibration curves, standard solutions (r2 > 0.99) in CH_3_CN/water, over the concentration range of 0.2–250 μM, were used. HPLC analyses were performed as described below.

#### 2.4.3. RP-HPLC Analysis

Quantitative analysis of **2e** and **1**, as the main metabolite, was performed using analytical RP-HPLC using Phenomenex Kinetex XB-C18 column (Phenomenex, Torrance, CA, USA, 5 μm, 4.6 × 250 mm). The column was connected to a Rheodyne model 7725 injector (Kyoto, Japan), a Shimadzu-10 ADsp HPLC system (Kyoto, Japan) and a Shimadzu SPD-20 A/SPD-20 AV UV-VIS detector (Kyoto, Japan). The mobile phase consisted of water 0.1% TFA (solvent A) and CH_3_CN 0.1% TFA (solvent B) at a constant flow rate of 1.0 mL/min. The injection volume was 20 µL, and gradient elution was employed from 30% to 100% solvent B over 15 min. The detection was carried out using a UV detector (Kyoto, Japan) set at 254 and 220 nm. Data acquisition and analysis were performed using standard chromatography software (Lab solutions LC, version 5.57 SP1, Shimadzu, Kyoto, Japan).

## 3. Results and Discussion

### 3.1. Synthesis and Chemical-Physical Characterization of Novel Compounds

#### 3.1.1. (S)-2-Isothiocyanato-N-propyl-4,5,6,7-tetrahydrobenzo[d]thiazol-6-amine Hydrochloride Salt (**2a**)

The mixture of (S)-N6-propyl-4,5,6,7-tetrahydrobenzo[d]thiazole-2,6-diamine (**1**, 500 mg, 3.4 mmol) and Na_2_CO_3_ (370 mg, 3.5 mmol) in acetone was placed in an ice-bath and thiophosgene (272 μL, 3.5 mmol) was added dropwise. The reaction mixture was kept at 0 °C for an hour and then at room temperature for an hour. The precipitated solid was filtered, and the solvent evaporated by distillation at reduced pressure. The obtained residue was treated with water and extracted with dichloromethane. The organic phases were dried with anhydrous Na_2_SO_4_, filtered and evaporated. The crude product was purified on a silica gel column using DCM/MeOH 9.5:0.5 (*v*/*v*) as the eluent mixture. The collected fractions were dried by distillation and the residue was crystallized with n-hexane, obtaining an orange solid product (**1a**). The compound **1a** was dissolved in anhydrous dichloromethane and HCl solution (2.0 M in diethyl ether) was added at 0 °C for one hour. The solvent was removed by vacuum distillation and the residue recrystallised with diethyl ether, obtaining a pure yellow solid (**2a**). Yield 20%. m.p. 109.7–110.4 °C. ESI-HRMS (M + H)^+^ *m*/*z* 253.07 calcd. for C_11_H_15_N_3_S_2_; found 254.1. ^1^H NMR (400 MHz, DMSO) δ 9.96 (s, 1H), 5.17 (s, 1H), 3.89–3.82 (ddd, J = 13.5, 8.1, 7.0 Hz, 2H), 3.48–3.41 (m, 1H) 2.84–2.80 (dd, J = 10.4, 5.7 Hz, 1H), 2.52–2.49 (m, 2H), 2.44–2.40 (m, 2H), 1.78–1.53 (m, 2H), 0.87 (t, J = 7.4 Hz, 3H). ^13^C NMR (101 MHz, DMSO) δ 198.67, 170.38, 101.00, 70.23, 59.01, 48.29, 38.26, 26.19, 20.11, 11.65.

#### 3.1.2. (S)-1-Allyl-3-(6-(propylamino)-4,5,6,7-tetrahydrobenzo[d]thiazol-2-yl)thiourea Hydrochloride Salt (**2b**)

A solution of (S)-N6-propyl-4,5,6,7-tetrahydrobenzo[d]thiazole-2,6-diamine (**1**, 200 mg, 0.946 mmol) in dry acetone (5 mL) was placed in a 10 mL closed reaction vessel and allylisothiocyanate (92 μL, 0.946 mmol) was added. The closed reaction vessel was placed in the cavity of a CEM microwave reactor, run under pressure and irradiated according to the following parameters: T, 70 °C; ramp time, 2 min; hold time, 10 min; pressure, 150 psi; power, 100 W. After cooling to 0 °C, the mixture was hydrolyzed with ice. The precipitate was washed with cold water and crude material was recrystallized with diethyl ether, providing a white solid product (**1b**). The obtained compound **1b** was dissolved in anhydrous dichloromethane and HCl solution (2.0 M in diethyl ether) was added at 0 °C for one hour. The solvent was removed by distillation at reduced pressure and the residue recrystallised with diethyl ether, obtaining a pure solid (**2b**). Yield 95%. m.p. 172.3–173.9 °C. ESI-HRMS (M + H)^+^ *m*/*z* 310.13 calcd. for C_14_H_22_N_4_S_2_; found 311.2. ^1^H NMR (400 MHz, DMSO-d6) δ 5.98 (ddd, J = 16.2, 10.9, 5.8 Hz, 1H), 5.72 (s, 1H), 5.49 (s, 1H), 5.23 (t, J = 14.9 Hz, 2H), 4.89 (s, 1H), 4.39 (m, 2H), 3.29 (m, 2H), 2.92 (dd, J = 15.0, 5.4 Hz, 1H), 2.74 (d, J = 6.5 Hz, 2H), 2.63–2.54 (m, 1H), 2.18 (s, 1H), 2.06–2.03 (m, 1H), 2.00–1.87 (m, 1H), 1.75–1.65 (m, 2H), 0.98 (t, J = 7.4 Hz, 3H). ^13^C NMR (101 MHz, DMSO-d6) δ 181.66, 165.80, 144.69, 134.17, 117.02, 115.93, 56.97, 48.65, 46.28, 30.94, 27.49, 26.19, 22.64, 11.55.

#### 3.1.3. (S)-1-Benzyl-3-(6-(propylamino)-4,5,6,7-tetrahydrobenzo[d]thiazol-2-yl)thiourea Hydrochloride Salt (**2c**)

The desired compound (**2c**) was prepared as described for **2b** starting from (S)-N6-propyl-4,5,6,7-tetrahydrobenzo[d]thiazole-2,6-diamine (**1**, 200 mg, 0.946 mmol) and benzyl isothiocyanate (125 μL, 0.946 mmol). Yield 91%. m.p. 155.4–156.3 °C. ESI-HRMS (M + H)^+^ *m*/*z* 360.14 calcd. for C_18_H_24_N_4_S_2_; found 361.2. ^1^H NMR (400 MHz, DMSO-d6) δ 9.37 (s, 1H), 8.10 (s, 1H), 7.32–7.26 (m, 4H), 7.21 (t, J = 5.7 Hz, 1H), 5.50 (s, 1H), 4.90–4.79 (qd, J = 15.4, 5.5 Hz, 2H), 2.74–2.53 (m, 4H), 2.52–2.49 (m, 2H), 2.04–1.93 (m, 2H), 1.92–1.88 (m, 1H), 1.66–1.49 (m, 2H), 0.87 (t, J = 7.2 Hz, 3H). ^13^C NMR (101 MHz, DMSO-d6) δ 181.65, 169.32, 140.44, 133.03, 128.49, 127.48, 126.86, 113.44, 55.97, 48.82, 25.83, 25.76, 23.20, 22.43, 11.47.

#### 3.1.4. (S)-1-Phenethyl-3-(6-(propylamino)-4,5,6,7-tetrahydrobenzo[d]thiazol-2-yl)thiourea Hydrochloride Salt (**2d**)

The desired compound (**2d**) was prepared as described for **2b** starting from (S)-N6-propyl-4,5,6,7-tetrahydrobenzo[d]thiazole-2,6-diamine (**1**, 200 mg, 0.946 mmol) and phenethyl isothiocyanate (141 μL, 0.946 mmol). Yield 93%. m.p. 125.4–126.9 °C. ESI-HRMS (M + H)^+^ *m*/*z* 374.16 calcd. for C_19_H_26_N_4_S_2_; found 375.2. ^1^H NMR (400 MHz, DMSO-d6) δ 9.36 (s, 1H), 7.55 (s, 1H), 7.30 (t, J = 7.4 Hz, 2H), 7.21 (dd, J = 13.9, 7.1 Hz, 3H), 5.45 (s, 1H), 3.79–3.76 (m, 2H), 3.41–3.66 (m, 1H), 2.88 (t, J = 7.6 Hz, 2H), 2.67–2.54 (m, 4H), 2.52–2.49 (m, 2H), 2.00–1.91 (m, 1H), 1.90–1.84 (m, 1H), 1.52–1.40 (m, 2H), 0.83 (t, J = 7.3 Hz, 3H). ^13^C NMR (101 MHz, DMSO-d6) δ 180.96, 169.32, 140.03, 133.03, 129.10, 128.82, 126.52, 113.46, 55.51, 47.28, 35.18, 25.79, 25.75, 23.20, 22.13, 11.48.

#### 3.1.5. 1-((3R,5R,7R)-Adamantan-1-yl)-3-((S)-6-(propylamino)-4,5,6,7-tetrahydrobenzo[d]thiazol-2-yl)thiourea Hydrochloride Salt (**2e** **-** **PRAM-ADA**)

The desired compound (**2e**) was prepared as described for **2b** starting from (S)-N6-propyl-4,5,6,7-tetrahydrobenzo[d]thiazole-2,6-diamine (**1**, 200 mg, 0.946 mmol) and 1-adamanthyl isothiocyanate (183 mg, 0.946 mmol). Yield 87%. m.p. 238.5–239.8 °C. ESI-HRMS (M + H)^+^ *m*/*z* 404.21 calcd. for C_21_H_32_N_4_S_2_; found 405.2. ^1^H NMR (400 MHz, DMSO-d6) δ 9.30 (s, 1H), 9.17 (s, 1H), 7.90 (s, 1H), 3.44 (s, 2H), 3.37–3.24 (m, 4H), 3.00 (dd, J = 15.6, 4.7 Hz, 1H), 2.90 (d, J = 5.0 Hz, 2H), 2.72 (dd, J = 15.3, 9.1 Hz, 1H), 2.61–2.57 (m, 1H), 2.25–2.21 (m, 1H), 2.06 (s, 2H), 1.96 (d, J = 2.5 Hz, 4H), 1.89 (ddd, J = 19.8, 11.4, 5.5 Hz, 2H), 1.73–1.66 (m, 2H), 1.62 (s, 4H), 0.93 (t, J = 7.4 Hz, 3H). ^13^C NMR (101 MHz, DMSO-d6) δ 179.56, 168.05, 139.80, 111.08, 59.23, 53.31, 46.37, 43.43, 35.23, 29.14, 25.58, 24.90, 23.19, 19.60, 11.55.

### 3.2. Amperometric H_2_S Releasing Properties

**Figure 1:**
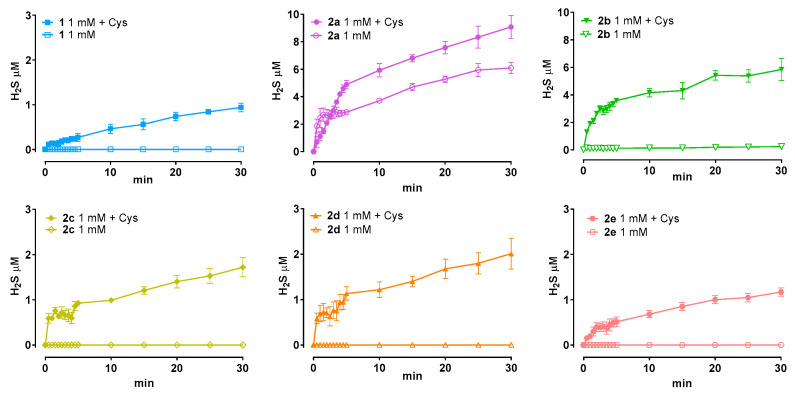
Amperometric time-dependent H_2_S release from the pramipexole-based H_2_S donor derivatives tested at 1 mM, in the presence and absence of L-cysteine (4 mM). Data are shown as mean ± SEM (n ≥ 6).

All pramipexole-based H_2_S-donor derivatives tested at 1 mM exhibited a time-dependent increase in H_2_S generation in the presence of L-cysteine, whereas negligible release was observed under cysteine-free conditions (Figure 1). The addition of L-cysteine markedly enhanced H_2_S production for all compounds, indicating a thiol-dependent activation mechanism. Under these conditions, most derivatives generated approximately 2 μM H_2_S, with compound **2b** reaching ~4 μM. In contrast, H_2_S levels remained essentially unchanged over time in the absence of L-cysteine, confirming that spontaneous hydrolysis or degradation does not significantly contribute to H_2_S formation.

Notably, compound **2a** displayed a distinct behavior compared to the other derivatives, releasing higher H_2_S levels both in the presence (~8 μM) and absence (~5 μM) of L-cysteine, suggesting a partial thiol-independent contribution to its H_2_S release. Overall, the concentration–time profiles obtained in the presence of L-cysteine were characterized by a progressive increase in H_2_S levels, further supporting the requirement of thiol availability to sustain efficient H_2_S donation for the majority of the tested compounds.

### 3.3. Measurement of Intracellular H_2_S Release in Murine Microglial Cells

**Figure 2:**
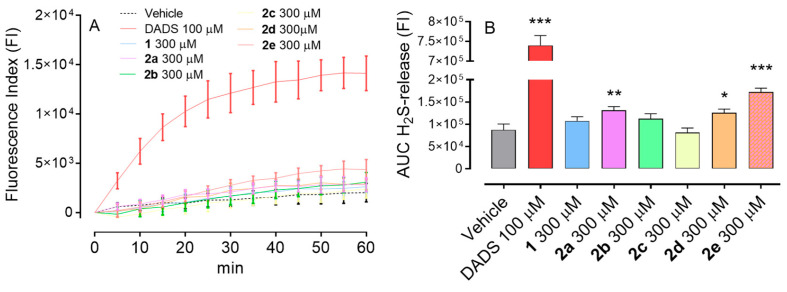
(**A**) Fluorescence index indicating the H_2_S release monitored for 60 min. (**B**) Quantification of the area under the curve (AUC) corresponding to H_2_S release. Data are expressed as mean ± SD (n ≥ 6). Statistical analysis was performed using one-way ANOVA followed by Bonferroni’s post hoc test versus vehicle. * indicates statistical significance vs. vehicle (* *p* < 0.05; ** *p* < 0.01; *** *p* < 0.001).

The evaluation of intracellular H_2_S release using BV2 cells revealed differences among the pramipexole-based derivatives tested at 300 μM. As shown in the kinetic curves (Figure 2A), compound **2e** displayed the most pronounced intracellular H_2_S increase during the time of incubation (60 min). Compounds **2a** and **2d** also elicited appreciable H_2_S accumulation, while the other derivatives produced moderate and not significant H_2_S release. When tested at 100 μM, none of the compounds induced appreciable intracellular H_2_S release.

The differences observed between the amperometric H_2_S measurements obtained in a cell-free system and those recorded in murine microglial cells primarily reflect the distinct nature of the experimental approaches rather than a divergence in donor behavior. The in vitro amperometric assay is designed to assess the intrinsic release kinetics of the compound in the presence of an excess of free thiols (obtained through the addition of L-cysteine), without biological interference. In contrast, measurements in microglial cells show H_2_S release within a complex intracellular environment, where the molecule is taken up and then releases H_2_S.

The AUC analysis (Figure 2B) further corroborated these findings. Compound **2e,** hereafter referred to as **PRAM-ADA**, showed the highest cumulative intracellular H_2_S exposure, identifying it as the most efficient donor of the series. Notably, compounds **2a** and **2d** also generated substantially higher AUC values compared with the other derivatives.

The concentrations used for the tested compounds were selected considering their distinct chemical properties, including sulfur content and solubility, and the interpretation of the results needs to consider this choice. In particular, DADS, employed as a positive control, contains two sulfur atoms and is known to release H_2_S efficiently under the experimental conditions. In contrast, the tested compounds exhibit different structural features and H_2_S-releasing capacities, which may require higher concentrations to achieve measurable biological effects. Furthermore, differences in solubility and stability in the experimental medium necessitated the use of increased concentrations for some compounds. Thus, the reported responses reflect compound-dependent activity under optimized experimental conditions rather than a direct comparison of potency on an equimolar basis.

### 3.4. Antioxidant Effect in an LPS-Induced BV-2 Model

Microglial cells play a central role in the progression of Parkinson’s disease by acting as key mediators of neuroinflammation and oxidative stress. Upon activation, microglia produce high levels of reactive oxygen species (ROS), nitric oxide, and pro-inflammatory cytokines, which contribute to dopaminergic neuronal dysfunction and degeneration [25]. Persistent microglial activation has been recognized as a major amplifier of neuronal injury in PD, linking neuroinflammation to oxidative damage within the nigrostriatal pathway. In this context, targeting microglial oxidative stress represents a complementary therapeutic strategy alongside dopaminergic modulation [26]. Notably, increasing evidence indicates that hydrogen sulfide (H_2_S) exerts regulatory effects on microglial function, attenuating activation and reducing the production of pro-inflammatory mediators and ROS [27]. H_2_S donors have been shown to suppress microglial activation and promote a shift toward a neuroprotective phenotype, thereby limiting inflammation-driven neuronal damage [28]. Therefore, the use of BV2 microglial cells in the present study is intended to evaluate the anti-inflammatory and antioxidant component of the hybrid compounds, while the pramipexole scaffold provides the dopaminergic pharmacological basis, supporting a dual-target strategy relevant to PD pathophysiology.

**Figure 3:**
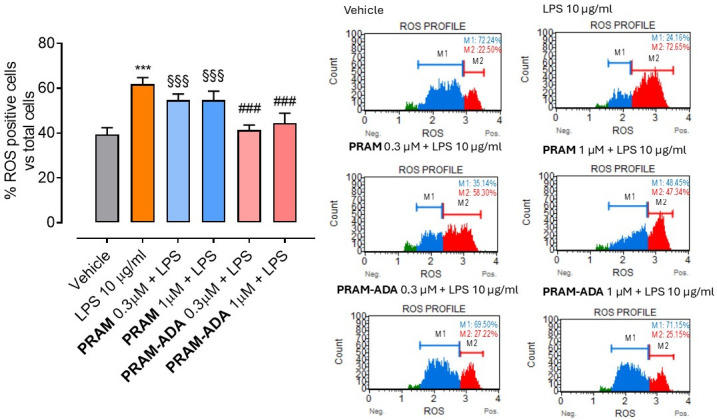
Quantification of ROS-+ cells following LPS-induced oxidative injury and treatment with **PRAM-ADA** hybrid compound. Data are expressed as mean ± SEM (n ≥ 6). Statistical significance (*p* < 0.05) is calculated using one-way ANOVA followed by Bonferroni post-hoc test. * indicates statistical significance vs. vehicle (*** *p* < 0.001); # vs. LPS (### *p* < 0.001); § vs. **PRAM-ADA** (§§§ *p* < 0.001). On the right, representative plots obtained for each treatment, through minicytofluorometric analysis. Green section represents cell debris. M1 indicates the population of ROS negative cells, while M2 ROS positive cells.

Thus, to investigate the potential protective properties of hybrid molecules combining dopaminergic activity with H_2_S-releasing features, an in vitro inflammatory model was employed for quantifying ROS levels through minicytofluorometric analysis, using LPS stimulation and BV-2 microglial cells. LPS treatment significantly increased the percentage of ROS-+ cells compared with vehicle. Treatment with **PRAM-ADA** hybrid compound, selected for further pharmacological evaluation based on its significant H_2_S-releasing properties, effectively attenuated ROS accumulation at both 0.3 and 1 μM, compared with vehicle. In contrast, the parent drug pramipexole (**PRAM**) did not produce a significant reduction in ROS at either concentration, highlighting that the antioxidant effect is due to the H_2_S-releasing modification (Figure 3). No decrease in cell viability was recorded (Supporting Information, Figure S2).

### 3.5. Evaluation of Anti-Senescence Effect in BV-2 Cells

**Figure 4:**
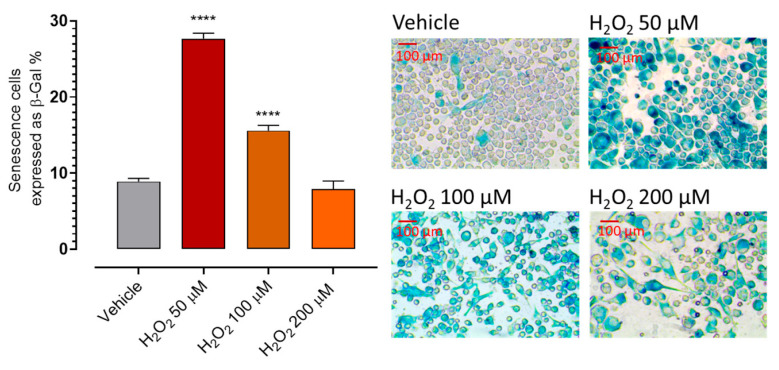
Set up of the H_2_O_2_-induced senescence model. Cells were treated with vehicle or increasing concentrations of H_2_O_2_ (50, 100, and 200 μM), and senescence was quantified by β-galactosidase staining and expressed as the percentage of β-Gal–positive cells. Data are expressed as mean ± SEM (n ≥ 6). Statistical significance (*p* < 0.05) is calculated using one-way ANOVA followed by Bonferroni post-hoc test. * indicates statistical significance vs. vehicle (**** *p* < 0.0001).

The senescence model was established in BV2 microglial cells. Senescent microglial cells are characterized by a pro-inflammatory and pro-oxidant phenotype, commonly referred to as the senescence-associated secretory phenotype (SASP), which promotes the release of cytokines, reactive oxygen species, and other mediators capable of exacerbating neuronal damage. The accumulation of such dysfunctional microglia has been implicated in amplifying chronic neuroinflammation and the progression of dopaminergic neurodegeneration. To establish and validate the oxidative stress–induced senescence model, cells were exposed to increasing concentrations of H_2_O_2_ (50, 100, and 200 μM) and senescence was evaluated by β-galactosidase staining (Figure 4). H_2_O_2_ 50 and 100 μM induced significant senescence, with the highest level of senescence observed at 50 μM, followed by a lower but still significant increase at 100 μM. Conversely, exposure to 200 μM H_2_O_2_ resulted in senescence levels comparable to the vehicle condition, suggesting that excessive oxidative stress compromised cell viability rather than promoting senescence (Supporting Information, Figure S3). Based on these results, 50 μM H_2_O_2_ was selected as the optimal concentration for inducing senescence.

**Figure 5:**
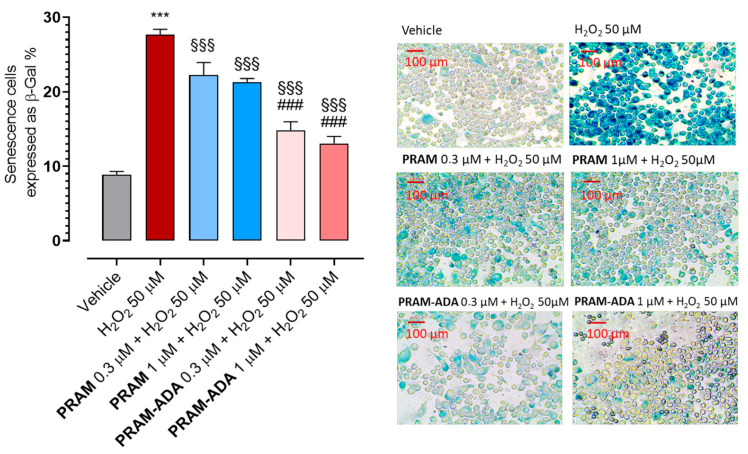
Quantification of senescent cells following H_2_O_2_-induced oxidative stress and treatment with **PRAM-ADA** hybrid compound. Cellular senescence was assessed by β-galactosidase staining and expressed as the percentage of β-Gal–positive cells relative to total cells. Data are expressed as mean ± SEM (n ≥ 6). Statistical significance (*p* < 0.05) is calculated using one-way ANOVA followed by Bonferroni post-hoc test. * indicates statistical significance vs. vehicle (*** *p* < 0.001); § vs. H_2_O_2_ (§§§ *p* < 0.001); # vs. **PRAM** (### *p* < 0.001). On the right, representative plots obtained for each treatment.

Pre-treatment with **PRAM-ADA** significantly reduced the percentage of senescent cells at both 0.3 and 1 μM (Figure 5). In contrast, the parent compound pramipexole (**PRAM**), which does not release H_2_S, produced only a modest, although significant, attenuation of H_2_O_2_-induced senescence.

These results suggest that controlled H_2_S release enhances the anti-senescent effects of pramipexole, supporting the concept that H_2_S supplementation may counteract oxidative stress–accelerated senescence in PD pathophysiology.

### 3.6. Chemical and Enzymatic Stability

The physicochemical properties of the selected hybrid compound, with particular focus on chemical and enzymatic stability, were assessed at different physiological pH conditions and in nutrient-rich Dulbecco’s Modified Eagle’s Medium (DMEM).

The chemical stability of **PRAM-ADA** was assessed at physiological pH (PBS, pH 7.4) and under acidic conditions representative of the gastric environment (simulated gastric fluid, SGF, pH 1.2), while enzymatic stability was investigated in a serum-containing culture medium (DMEM supplemented with fetal bovine serum, FBS) to evaluate its behavior under physiologically relevant conditions. The concentrations of **PRAM-ADA** and parent compound derived from the hydrolytic cleavage (pramipexole) were quantified using RP-HPLC at definite time points.

When incubated in PBS at pH 7.4 and 37 °C, **PRAM-ADA** exhibited moderate stability, with a calculated half-life (t_1_/_2_) of 4.8 h. This result indicates that the molecule undergoes gradual degradation at physiological pH, suggesting a limited but measurable susceptibility to hydrolytic processes in an aqueous buffered environment. In contrast, a faster degradation was observed under acidic conditions; indeed, in SGF (pH 1.2), the compound showed a reduced half-life of 1.7 h, highlighting an increased instability in a strongly acidic medium.

The enzymatic stability was evaluated in Dulbecco’s Modified Eagle Medium (DMEM) supplemented with fetal bovine serum (FBS), a medium rich in proteins and enzymes that better approximates the biological environment encountered in vitro and, to some extent, in vivo. Under these conditions, **PRAM-ADA** displayed a half-life of 1.8 h, comparable to that observed in SGF. This finding indicates a pronounced susceptibility to enzymatic or protein-mediated degradation, likely due to the presence of serum hydrolytic enzymes.

## 4. Conclusions

The present study describes the design and pharmacological characterization of a new class of pramipexole-derived H_2_S-releasing hybrids aimed at addressing the major molecular and cellular processes implicated in PD. Herein, a thiourea group was employed for the first time as an H_2_S-donating linker. By integrating a dopaminergic pharmacophore with H_2_S-donating moieties, the designed molecules were developed to widen the therapeutic profile of pramipexole beyond dopamine receptor agonism.

Recent evidence supports a central neuroprotective role of H_2_S in Parkinson’s disease, particularly through the modulation of oxidative stress, mitochondrial dysfunction, neuroinflammation, and cell death pathways. In this study, the pramipexole-based hybrid **2e** (**PRAM-ADA**) emerged as the most effective intracellular H_2_S donor, displaying a sustained and thiol-dependent release profile associated with enhanced antioxidant and anti-senescent effects in microglial cells. Notably, **PRAM-ADA** significantly attenuated ROS accumulation and cellular senescence, key contributors to neuroinflammation and disease progression, outperforming pramipexole alone and highlighting the added value of H_2_S donation. Furthermore, its moderate stability under physiological conditions, combined with faster degradation in the biological environment, supports its potential as a dual-acting dopaminergic/H_2_S-releasing agent.

Although the findings reported here are highly encouraging, further investigations are required to validate these results in more physiologically relevant settings. In this regard, the intracellular release studies were performed in microglial cells, and additional analyses in dopaminergic neurons and astrocytes will be necessary to fully define the neuroprotective potential of these compounds. Despite these considerations, the current findings provide strong proof-of-concept for the therapeutic relevance of coupling dopaminergic agonism with H_2_S-based cytoprotection.

In conclusion, this study highlights the translational potential of pramipexole-derived H_2_S donors as innovative, multi-target agents for PD. By leveraging the distinct yet complementary actions of H_2_S and dopamine receptor modulation, these hybrid molecules may represent a new generation of multitarget neuroprotective agents.

## Data Availability

The original contributions presented in this study are included in the article/Supplementary Materials. Further inquiries can be directed to the corresponding author.

## Supplementary Materials

The following supporting information can be downloaded at: https://www.mdpi.com/article/10.3390/antiox15050628/s1, Figure S1: Toxicity of PRAM and PRAM-ADA; Figure S2: Cell viability after LPS treatment; Figure S3: Cell viability following senescence protocol.

## Abbreviations

The following abbreviations are used in this manuscript:

PD	Parkinson’s disease
H_2_S	Hydrogen sulfide
ITCs	Isothiocyanates
ROS	Reactive Oxygen Species
MTDLs	Multitarget-directed ligands
TLC	Thin-layer chromatography
FTMS	Fourier transform mass spectrometer
AUC	Area under the curve
DMEM	Dulbecco’s Modified Eagle Medium
FBS	Fetal Bovine Serum
SGF	Simulated gastric fluid
PRAM	Pramipexole

## References

[1] Poewe W., Seppi K., Tanner C., Halliday G.M., Brundin P., Volkmann J., Schrag A.-E., Lang A.E. (2017). Parkinson disease. Nat. Rev. Dis. Prim..

[2] Morris H.R., Spillantini M.G., Sue C.M., Williams-Gray C.H. (2024). The pathogenesis of Parkinson’s disease. Lancet.

[3] Olanow C.W., Stern M.B., Sethi K. (2009). The scientific and clinical basis for the treatment of Parkinson disease. Neurology.

[4] Chakrabarti S., Bisaglia M. (2023). Oxidative Stress and Neuroinflammation in Parkinson’s Disease: The Role of Dopamine Oxidation Products. Antioxidants.

[5] Dong-Chen X., Yong C., Yang X., Chen-Yu S., Li-Hua P. (2023). Signaling pathways in Parkinson’s disease: Molecular mechanisms and therapeutic interventions. Signal Transduct. Target. Ther..

[6] Kimura H. (2002). Hydrogen sulfide as a neuromodulator. Mol. Neurobiol..

[7] Panthi S., Hyung-Joo C., Junyang J., Na Young J. (2016). Physiological Importance of Hydrogen Sulfide: Emerging Potent Neuroprotector and Neuromodulator. Oxid. Med. Cell. Longev..

[8] Dogaru B.G., Munteanu C. (2023). The Role of Hydrogen Sulfide (H_2_S) in Epigenetic Regulation of Neurodegenerative Diseases: A Systematic Review. Int. J. Mol. Sci..

[9] Hu L.F., Lu M., Tiong C.X., Dawe G.S., Hu G., Bian J.S. (2010). Neuroprotective effects of hydrogen sulfide on Parkinson’s disease rat models. Aging Cell.

[10] Vandiver M.S., Paul B.D., Xu R., Karuppagounder S., Rao F., Snowman A.M., Seok Ko H., Lee Y.I., Dawson V.L., Dawson T.M. (2013). Sulfhydration mediates neuroprotective actions of parkin. Nat. Commun..

[11] Sita G., Hrelia P., Tarozzi A., Morroni F. (2016). Isothiocyanates Are Promising Compounds against Oxidative Stress, Neuroinflammation and Cell Death that May Benefit Neurodegeneration in Parkinson’s Disease. Int. J. Mol. Sci..

[12] Hacet F., Becer E., Vatansever H.S., Yücecan S. (2023). Investigation of Neuroprotective Effects of Sulforaphane and Allyl Isothiocyanate in an in vitro Alzheimer’s Disease Model. Pharmacogn. Mag..

[13] Olayanju J.B., Bozic D., Naidoo U., Sadik O.A. (2024). A Comparative Review of Key Isothiocyanates and Their Health Benefits. Nutrients.

[14] Caglayan B., Kilic E., Dalay A., Altunay S., Tuzcu M., Erten F., Orhan C., Gunal M.Y., Yulug B., Juturu V. (2019). Allyl isothiocyanate attenuates oxidative stress and inflammation by modulating Nrf2/HO-1 and NF-κB pathways in traumatic brain injury in mice. Mol. Biol. Rep..

[15] Lin Y., Yang X., Lu Y., Liang D., Huang D. (2019). Isothiocyanates as H_2_S Donors Triggered by Cysteine: Reaction Mechanism and Structure and Activity Relationship. Org. Lett..

[16] Scognamiglio A., Cerqua I., Citi V., Martelli A., Spezzini J., Calderone V., Rimoli M.G., Sodano F., Caliendo G., Santagada V. (2024). Isothiocyanate-Corticosteroid Conjugates against asthma: Unity makes strength. Eur. J. Med. Chem..

[17] Sestito S., Daniele S., Pietrobono D., Citi V., Bellusci L., Chiellini G., Calderone V., Martini C., Rapposelli S. (2019). Memantine prodrug as a new agent for Alzheimer’s Disease. Sci. Rep..

[18] Sestito S., Cirone I., Sagona S., Runfola M., Raffellini L., La Rocca V., Citi V., Martelli A., Daniele S., Lai M. (2025). Design, synthesis and biological evaluation of new H_2_S-releasing rivastigmine derivatives as neuroprotective molecules. Eur. J. Med. Chem..

[19] Mierau J., Schneider F.J., Ensinger H.A., Chio C.L., Lajiness M.E., Huff R.M. (1995). Pramipexole binding and activation of cloned and expressed dopamine D2, D3 and D4 receptors. Eur. J. Pharmacol..

[20] Zaorska E., Hutsch T., Gawryś-Kopczyńska M., Ostaszewski R., Ufnal M., Koszelewski D. (2019). Evaluation of thioamides, thiolactams and thioureas as hydrogen sulfide (H_2_S) donors for lowering blood pressure. Bioorg. Chem..

[21] Citi V., Martelli A., Bucci M., Piragine E., Testai L., Vellecco V., Cirino G., Calderone V. (2020). Searching for novel hydrogen sulfide donors: The vascular effects of two thiourea derivatives. Pharmacol. Res..

[22] Verhagen Metman L., Del Dotto P., van den Munckhof P., Fang J., Mouradian M.M., Chase T.N. (1998). Amantadine as treatment for dyskinesias and motor fluctuations in Parkinson’s disease. Neurology.

[23] Benavides G.A., Squadrito G.L., Mills R.W., Patel H.D., Isbell T.S., Patel R.P., Darley-Usmar V.M., Doeller J.E., Kraus D.W. (2007). Hydrogen sulfide mediates the vasoactivity of garlic. Proc. Natl. Acad. Sci. USA.

[24] Itahana K., Campisi J., Dimri G.P. (2007). Methods to detect biomarkers of cellular senescence: The senescence-associated beta-galactosidase assay. Methods Mol. Biol..

[25] Peterson L.J., Flood P.M. (2012). Oxidative stress and microglial cells in Parkinson’s disease. Mediat. Inflamm..

[26] Mosley R.L., Benner E.J., Kadiu I., Thomas M., Boska M.D., Hasan K., Laurie C., Gendelman H.E. (2006). Neuroinflammation, Oxidative Stress and the Pathogenesis of Parkinson’s Disease. Clin. Neurosci. Res..

[27] Zhou L., Wang Q. (2023). Advances of H_2_S in Regulating Neurodegenerative Diseases by Preserving Mitochondria Function. Antioxidants.

[28] Tian Q., Tang H.L., Tang Y.Y., Zhang P., Kang X., Zou W., Tang X.Q. (2022). Hydrogen Sulfide Attenuates the Cognitive Dysfunction in Parkinson’s Disease Rats via Promoting Hippocampal Microglia M2 Polarization by Enhancement of Hippocampal Warburg Effect. Oxid. Med. Cell. Longev..

