# Supplementary material for: Thiourea-Based H2S-Releasing Pramipexole Hybrids as Neuroprotective Agents
Source: Antioxidants (Basel). 2026 May 15;15(5):628. doi: 10.3390/antiox15050628 (PMC13203430; doi:10.3390/antiox15050628)
Supplement: Supplementary file 1 [file antioxidants-15-00628-s001.zip › antioxidants-4227897_supplementary.pdf]

Supporting information

WST-1 cell viability assay

Safety profile of PRAM and PRAM-ADA

BV-2 cells were seeded in a clear 96-well plate at a density of 10×10^3 per well. After 24 h, the culture medium was replaced with 90 µL of fresh medium, and vehicle (DMSO 1%), PRAM (0.3 µM - 300µM) or PRAM-ADA (0.3 µM - 300 µM) were added for 24 h. Water-soluble tetrazolium salt-1 (WST-1, Roche, Basel, Switzerland) was incubated in each well (1:10) at 37 °C in a CO_2 (5%) incubator for 1 h. Cell viability was spectrophotometrically assessed at $\lambda = 495$ nm using the multiwell plate reader EnSpire (PerkinElmer, Waltham, MA, USA) and expressed as % vs vehicle.

Cytotoxicity of the experimental conditions

Cytotoxicity of the treatments used for ROS assessment and senescence model was determined by exposing cells to the indicated treatments for time corresponding to those employed in the respective assays, reproducing the same conditions. Then, Water Soluble Tetrazolium – 1 (WST-1, Merck KGaA, Germany), an absorbance probe which is converted into formazan salts by metabolically active cells, was added and incubated at 37°C and 5% CO2 for 1 h. At the end of the experimental procedure, the absorbance was read at 450 nm with EnSpire® (Perkin-Elmer, USA) spectrophotometer and the results analyzed by GraphPad Prism.

Results

Safety profile of PRAM and PRAM-ADA

Cell viability remained above ~80% under all experimental conditions after 24 h of treatment with PRAM and PRAM-ADA at the tested concentrations. A slight reduction in viability was observed at higher concentrations; however, no significant cytotoxic effects were detected compared to control conditions (Figure S1).

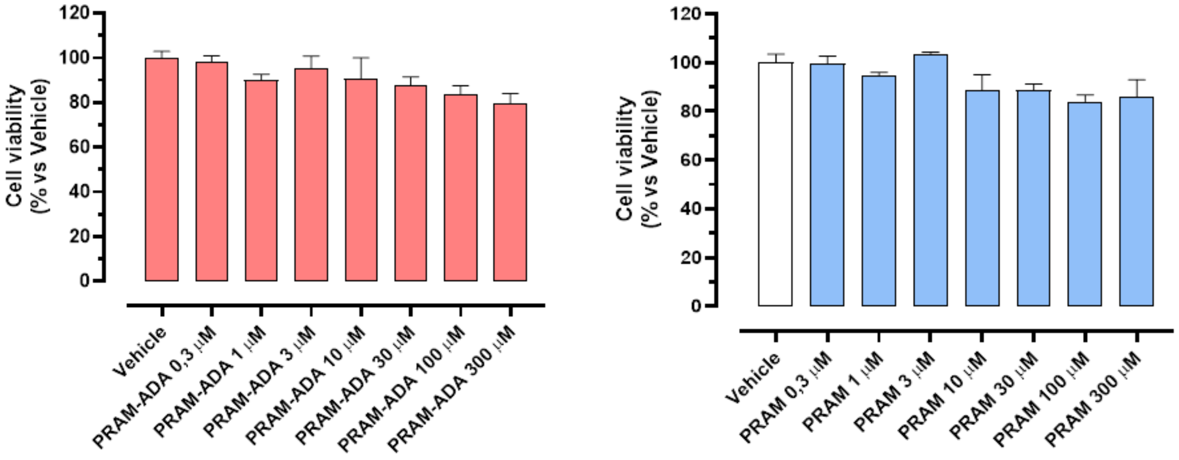

Figure S1 – Toxicity of PRAM and PRAM – ADA. The graphs represent the percentage of cell viability in different experimental conditions. The vehicle-treated group is set as 100% of cell viability. Data are presented as mean ± SEM (n > 6), with statistical significance calculated using One way ANOVA, followed by Bonferroni post-test.

<u>*Cytotoxicity of the experimental conditions*</u>

LPS induced did not show reduction in cell viability after 24 h. Co-treatment with PRAM or PRAM-ADA (0.3 and 1 µM) did not exacerbate cytotoxicity, maintaining viability close to control levels (Figure S2).

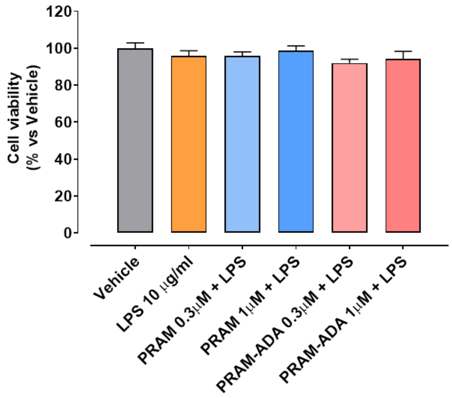

Figure S2 – Cell viability after LPS treatment. The graph represents the percentage of cell viability in different experimental conditions. The vehicle-treated group is set as 100% of cell viability. Data are presented as mean ± SEM (n > 6), with statistical significance calculated using One way ANOVA, followed by Bonferroni post-test.

H_2O_2 induced a concentration-dependent decrease in cell viability, with significant reduction at 100 µM and a marked cytotoxic effect at 200 µM. H_2O_2 (50 µM) induced a modest and not significant

reduction in cell viability after 24 h. Co-treatment with PRAM or PRAM-ADA (0.3 and 1 μM) did not exacerbate cytotoxicity, maintaining viability close to control levels (Figure S3).

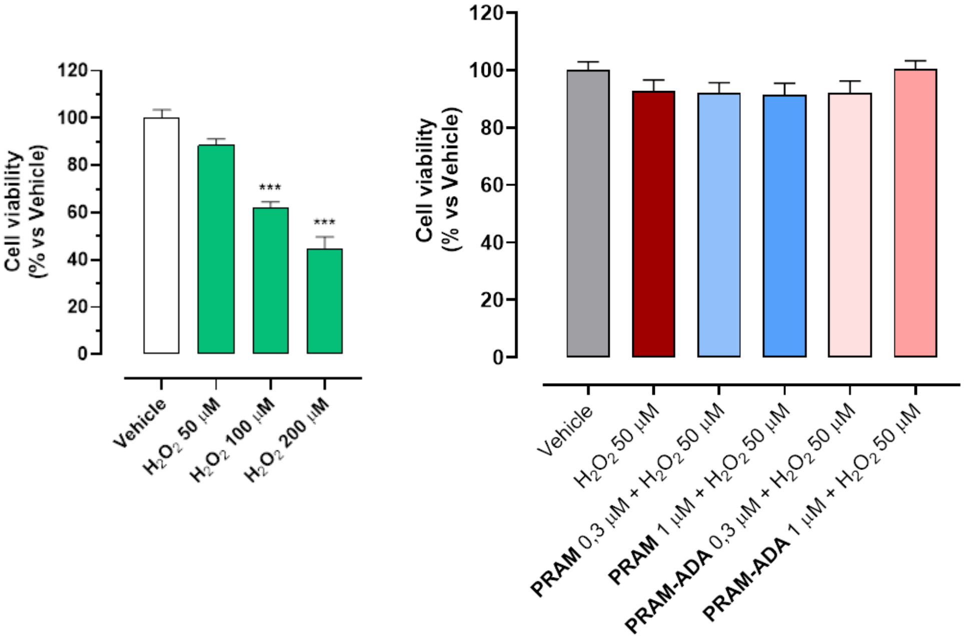

Figure S3 – Cell viability following senescence protocol. The graphs represent the percentage of cell viability in different experimental conditions. The vehicle-treated group is set as 100% of cell viability. Data are presented as mean ± SEM (n > 6), with statistical significance calculated using One way ANOVA, followed by Bonferroni post-test.
